# Biting behaviour, spatio-temporal dynamics, and the insecticide resistance status of malaria vectors in different ecological zones in Ghana

**DOI:** 10.1186/s13071-023-06065-9

**Published:** 2024-01-09

**Authors:** Osei K. Akuoko, Shittu B. Dhikrullahi, Isaac A. Hinne, Abdul R. Mohammed, Christopher M. Owusu-Asenso, Sylvester Coleman, Samuel K. Dadzie, Rosina Kyerematen, Daniel A. Boakye, Michael D. Wilson, Yaw A. Afrane

**Affiliations:** 1 Department of Parasitology, Noguchi Memorial Institute for Medical Research, College of Health Sciences, University of Ghana, Legon, Accra, Ghana; 2 African Regional Post-Graduate Programme in Insect Science, College of Basic and Applied Science, University of Ghana, Legon, Accra, Ghana; 3 Department of Medical Microbiology, Centre for Vector-Borne Diseases Research, University of Ghana Medical School, University of Ghana, Korle-Bu, Accra, Ghana; 4 Department of Animal Biology and Conservation Science, College of Basic and Applied Sciences, University of Ghana, Legon, Accra, Ghana; 5 Department of Clinical Microbiology - Vector Biology Laboratory, School of Medicine and Dentistry (SMD)-College of Health Sciences, Kwame Nkrumah University of Science and Technology (KNUST), Kumasi, Ghana; 6 Department of Biochemistry and Molecular Biology, CABNR, University of Nevada, Reno, NV USA

**Keywords:** *Anopheles*, Indoor and outdoor densities, Biting time, Sporozoite rate, Genotypic resistance

## Abstract

**Background:**

A significant decrease in malaria morbidity and mortality has been attained using long-lasting insecticide-treated nets and indoor residual spraying. Selective pressure from these control methods influences changes in vector bionomics and behavioural pattern. There is a need to understand how insecticide resistance drives behavioural changes within vector species. This study aimed to determine the spatio-temporal dynamics and biting behaviour of malaria vectors in different ecological zones in Ghana in an era of high insecticide use for public health vector control.

**Methods:**

Adult mosquitoes were collected during the dry and rainy seasons in 2017 and 2018 from five study sites in Ghana in different ecological zones. Indoor- and outdoor-biting mosquitoes were collected per hour from 18:00 to 06:00 h employing the human landing catch (HLC) technique. Morphological and molecular species identifications of vectors were done using identification keys and PCR respectively. Genotyping of insecticide-resistant markers was done using the TaqMan SNP genotyping probe-based assays. Detection of *Plasmodium falciparum* sporozoites was determined using PCR.

**Results:**

A total of 50,322 mosquitoes belonging to four different genera were collected from all the study sites during the sampling seasons in 2017 and 2018. Among the Anophelines were *Anopheles gambiae* s.l. 93.2%, (31,055/33,334), *An*. *funestus* 2.1%, (690/33,334), *An*. *pharoensis* 4.6%, (1545/33,334), and *An*. *rufipes* 0.1% (44/33,334). Overall, 76.4%, (25,468/33,334) of *Anopheles* mosquitoes were collected in the rainy season and 23.6%, (7866/33,334) in the dry season. There was a significant difference (*Z* = 2.410; *P* = 0.0160) between indoor-biting (51.1%; 15,866/31,055) and outdoor-biting *An. gambiae* s.l. (48.9%; 15,189/31,055). The frequency of the Vgsc-1014F mutation was slightly higher in indoor-biting mosquitoes (54.9%) than outdoors (45.1%). Overall, 44 pools of samples were positive for *P. falciparum* CSP giving an overall sporozoite rate of 0.1%.

**Conclusion:**

*Anopheles gambiae* s.l. were more abundant indoors across all ecological zones of Ghana. The frequency of G119S was higher indoors than outdoors from all the study sites, but with higher sporozoite rates in outdoor mosquitoes in Dodowa and Kpalsogu. There is, therefore, an urgent need for a supplementary malaria control intervention to control outdoor-biting mosquitoes.

**Graphical Abstract:**

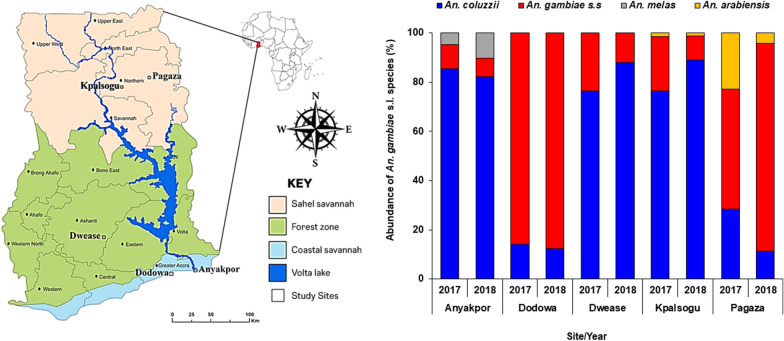

**Supplementary Information:**

The online version contains supplementary material available at 10.1186/s13071-023-06065-9.

## Background

The malaria burden in Africa is generally attributed to the relatively effective vector system that is made up of *Anopheles gambiae* s.l. and *An. funestus* [1, 2]. The transmission potential of these vectors varies between climatic seasons, ecological zones, and sometimes among areas in close proximity [3, 4]. In Ghana, studies have shown that *An. gambiae* s.l. and *An. funestus* are the predominant malaria vectors and they occur in sympatry over much of their range [5, 6]. Information on the various malaria vector species and their distribution under diverse ecological conditions are essential in their control strategies [7, 8].

There are three main ecological zones in Ghana: the coastal savannah zone in the south, the forest zone in the middle part, and the Sahel savannah zone in the northern part of Ghana. There are also transition areas between these zones. The coastal and forest zones have a bimodal rainfall pattern which allows for two peaks of malaria transmission, while the Sahel savannah zone has unimodal rainfall pattern which makes malaria transmission seasonal. The different climatic conditions experienced in the various ecological areas contribute to differences in malaria vector species composition, hence malaria transmission [9, 10].

Vector control is key in the malaria control strategy [11], and like many other African countries, long-lasting insecticide-treated nets (LLINs) and indoor residual spraying (IRS) are used in Ghana [12]. These methods, which target only indoor-biting mosquitoes, have achieved remarkable successes towards malaria elimination but the progress of these achievements has plateaued [12] because of the development and fast spread of insecticide resistance. The success of IRS and LLINs is largely based on the anthropophilic, endophagic, and endophilic behaviours of *Anopheles* vectors; however, there is a growing threat of both physiological and behavioural resistance to the insecticides used in these vector control strategies [13, 14]. The complexity of controlling malaria is attributed to changes in species and the behavioural pattern of the malaria vectors [13, 15–18] and these variations in mosquito bionomics are attributed to the possible influence of IRS and LLINs and the development of insecticide resistance [19, 20]. The development of resistance by these vectors may maintain transmission where control interventions have been successful [21]. The wide and prolonged use of LLINs and IRS causes certain behavioural changes in *Anopheles* vectors that help them circumvent or eschew insecticide-treated areas [13, 22]. The most principal of such adaptations includes a change in feeding behaviour.

*Anopheles* mosquitoes tend to change from their historically late night indoor-biting to early night and outdoor-biting times [13]. These mosquitoes avoid IRS and LLINs control by feeding and resting outdoors. They also feed in the early hours of the evening when people are outside and not in bed and/or early in the morning when people are out of their bed nets [13, 14, 23]. The local dynamics of insecticide resistance may be impacted by the spatio-temporal variation in insect vectors [24] albeit selection pressure may have resulted in the variations in mechanism of insecticide resistance in malaria vectors [24]. Studies have shown strong association between observed frequency of knock-down resistance (*kdr*) mutations and acetylcholine esterase (*Ace-1*) and resistance to pyrethroids and DDT in field mosquito populations [25, 26]; therefore, the presence of either *kdr* or *Ace-1* gene in a field population of mosquitoes is a reliable indicator of both resistance prevalence and high individual resistance [27].

The variations in species distribution in different ecological zones could be influenced by some landscape barriers to gene flow and exposure of the vector population to different levels of insecticide pressures [28]. Mutations caused by these factors at the neurons might be having a pleiotropic effect on the mosquito behaviour. Therefore, this study was to determine the biting behaviour, spatio-temporal dynamics, and insecticide resistance status of malaria vectors in different ecological zones in Ghana in an era of high insecticide use for public health vector control. This information will provide a better understanding of how the ecology in Ghana affects vector seasonal dynamics and explain the interactions between increased insecticide resistance in the malaria vector population and the ensuing biting behaviour patterns.

## Methods

### Study sites

**Fig. 1:**
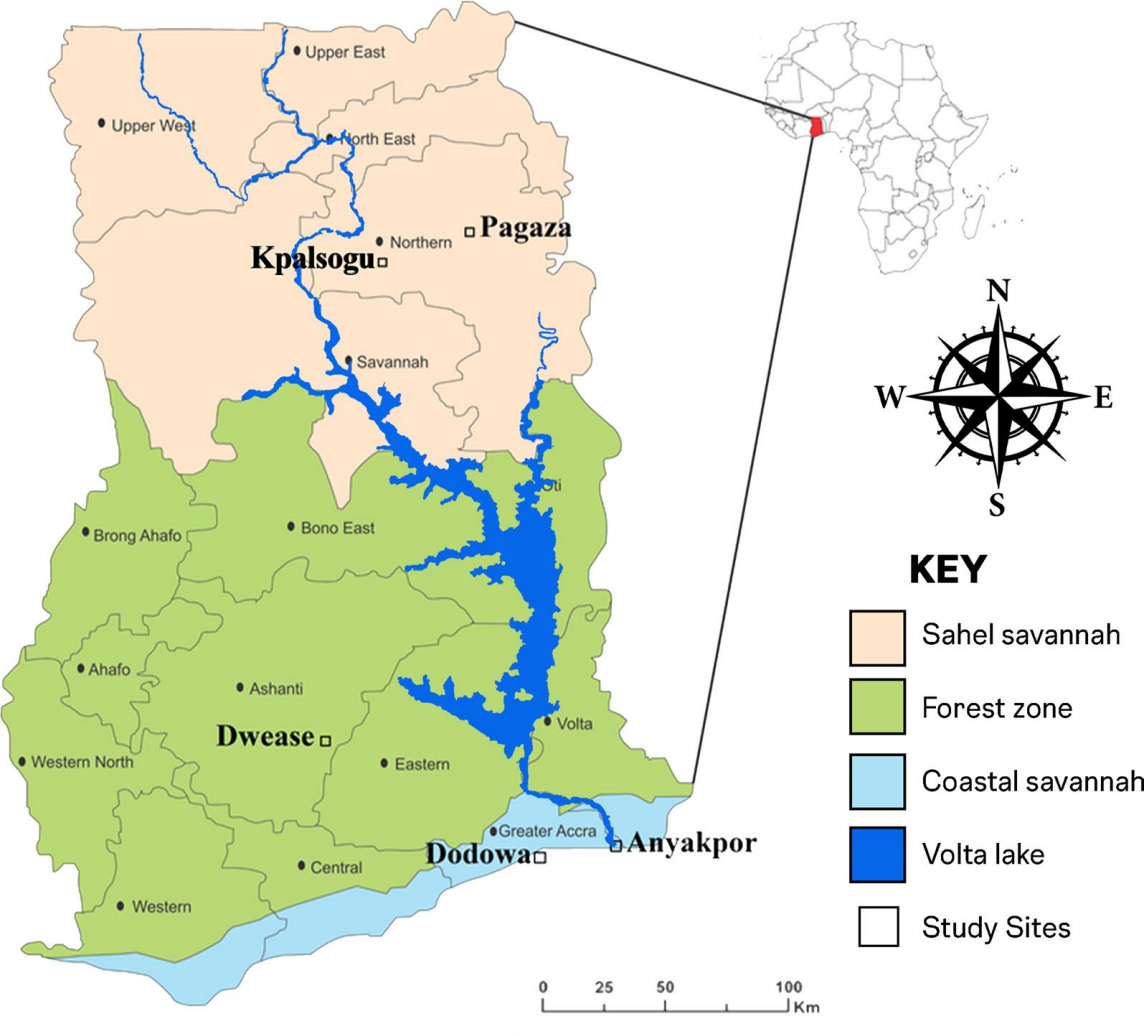
A map of Ghana showing the various study sites

Adult mosquitoes were collected during the dry season (February–March) and the rainy season (May–July) in 2017 and 2018 from five study sites in Ghana. The study sites were selected from four ecological zones: Anyakpor (5° 46′ 51.96″ N 0° 35′ 12.84″ E) in the coastal savannah zone; Dodowa (5° 52′ 58.3212'' N 0° 5′ 52.9548″ W) in the coastal-forest transition zone; Dwease (6° 32′ 3.05 "N 1° 14′ 42.22″ W) in the forest zone; Pagaza (9° 22′ 33.34″ N 0° 42′ 29.67″ W) and Kpalsogu (9° 33′ 45.2″ N 1° 01′ 54.6″ W) both in the Sahel savannah zone. These sites are shown in Fig. 1.

Anyakpor is a rural coastal community in the Ada East District of Ghana. It has a dry equatorial climate with temperatures between 23°C – 33°C and a bimodal rainfall pattern with a long rainy season from April to June and a short rainy season from October to November. Farming activities occur all year round, supported by an irrigation scheme. This allows for uninterrupted farming activities throughout the year. There are dug-out wells and other water impoundments which collect water during the dry and rainy season, and these may serve as suitable breeding sites and eventually affect the densities of mosquitoes. A previous study by Hinne et al. [29], reported that the most dominant species present in this site was *Anophelescoluzzii* [29].

Dodowa is a town in the Shai Osudoku district with an average temperature of 27 ℃ and a bimodal rainfall pattern like Anyakpor. It has a secondary forest-type vegetation with little original virgin forest left because of deforestation. Dwease is also a rural community close to Dwease in the Asante-Akim Central municipality with a wet-semi equatorial climate characterized by bimodal rainfall just like Anyakpor. Dwease has a semi-deciduous forest vegetation of open and closed forests.

Kpalsogu and Pagaza are rural communities in the Kumbungu and Tamale municipalities respectively. They have a unimodal rainfall pattern from May to November and a long dry season from December to April. The mean annual temperature is around 28 °C but can get to a maximum of 42 °C. Kpalsogu is close to a dam linked to an irrigation scheme which allows uninterrupted farming activities throughout the year. There are other water impoundments which collect water during the rainy season for irrigation in the dry season. In the rainy season, these dams overflow, creating many swamps which are suitable breeding habitats for *Anopheles* mosquitoes. Water from the dam which is diverted through canals to farms also provides breeding sites for mosquitoes and may affect mosquito densities within the area. There is also an IRS campaign supported by the President’s Malaria Initiative (PMI) to prevent malaria ongoing within this community. Exposure of mosquitoes to sublethal doses of insecticide may facilitate resistance within the vector population. Moreover, this indoor vector control strategy may facilitate outdoor biting by the malaria vectors present there.

### Mosquito collections

Indoor- and outdoor-biting mosquitoes were collected per hour from 18:00 to 06:00 h for four different nights in four houses per season by a two-person team of trained catchers in eight randomly selected houses employing the human landing catch (HLC) technique [30]. The study design ensured that randomly selected houses had household members sleeping under bednets or were covered under IRS for vector control. This was to ensure that there was no bias in selective factors such as IRS and presence of bednets. Collections were done in the five sentinel sites over four ecological zones of Ghana. Each study site was sectioned into four to ensure a fair representation of the mosquito population at the study site. Briefly, volunteers sat in the dark with their lower limbs exposed and, with the aid of a flashlight, located and collected the blood-seeking mosquitoes with a collection tube when they landed in search of a blood meal. Indoor and outdoor collectors were rotated hourly to avoid differences in individual attractiveness or repulsiveness to mosquitoes and as a precaution against dozing. Outdoor human biting catches were carried out at the same household 10 m away [31]. Independent staff supervised rotations and regularly walked between different groups for whole-night quality control of collectors placed inside and outside dwellings.

Hourly mosquitoes caught were killed by placing them in the − 20°C freezer for 15 min or chloroform for 1 min and then kept separately in individual tubes containing silica gel, pre-labelled with date, time, and location of capture, and taken to the laboratory for identification [32]. Mosquito biting pattern was classified as follows: early evening (EE) (18:00–22:00 h), late evening (LE) (22:00–4:00 h), and early morning (EM) (4:00–6:00 h).

### Morphological and molecular identification of vector mosquitoes

Mosquitoes collected were identified morphologically using a simplified key adopted from Gillies and Coetzee [33]. A sub-sample from the total *An. gambiae* s.l. collected over the entire period was selected according to study site, season, and location (indoor or outdoor) in a proportion of 10%. This was used to further discriminate members of the *An. gambiae* complex by PCR and RFLP-PCR. The legs of each mosquito were used for DNA extraction as previously described by Scott et al. [34]. Four sets of primers (*Anopheles gambiae, An. arabiensis, An. melas*, and universal primer) were used in PCR for the identification of members of the *An. gambiae* s.l. species complex [34]. *Anopheles gambiae* s.s. and *An. coluzzii* were distinguished by PCR-RFLP using the method of Fanello et al. [35].

### Genotyping for insecticide resistance mutations

Genomic DNA extracted from the legs of the indoor and outdoor mosquito samples were used to detect the presence of insecticide resistance mutations using a TaqMan SNP genotyping probe-based assay [36]. These markers include Vgsc-1014F and Vgsc-1014S. The same set of samples was also genotyped for Ace1-119S mutation [36].

### Detection of sporozoite

The heads and thoraces of mosquito samples were used to detect the presence of *Plasmodium falciparum* sporozoite using polymerase chain reaction (PCR) as described by Echeverry et al. [37]. Twenty mosquitoes were pooled according to the site, species, and collection time for the detection of sporozoite; a total of 643 pools were constituted from 12,860 *An. gambiae* s.l. including those used for species identification. Pooling of mosquito samples was done for logistic reasons to minimize reagent consumption.

### Data analysis

Descriptive analysis was performed to compare the abundance of malaria vectors in the different study sites (ecological zones), seasons, biting locations, and biting times. The chi-square and Fisher's exact tests were used to test the association between two categorical variables. The Mann-Whitney U and Kruskal-Wallis tests were used to test the associations between continuous and categorical variables. Generalized linear mixed model was used to model the effects of mosquito behaviour, season, and sampling period on *Anopheles* mosquito abundance. All statistical analyses were conducted in STATA version 15 software (StataCorp. 2017. Stata Statistical Software: Release 15. College Station, TX: StataCorp LLC). Alpha level was set at 0.05 and the proportions were estimated with confidence intervals in R (v 4.3.1). The sporozoite infection rate (IR) expressed as the proportion of mosquitoes positive for *Plasmodium* sporozoite was calculated according to the method previously described by Maia et al*.* [38]. The *kdr* L1014F and *Ace 1* G119S mutation frequencies were calculated according to the following formula:

$$F (kdr) = (2RR + RS)/2n$$

where RR is the number of homozygotes, RS is the number of heterozygotes, and *n* is the total number of specimens analysed.

## Results

### Abundance and seasonal distribution of malaria vectors

Overall, a total of 50,322 mosquitoes belonging to three different genera were collected from all the study sites during the two sampling seasons. In general, more mosquitoes were collected in 2017 (*n* = 26,415; 95% CI = 0.52–0.53) than in 2018 (*n* = 23,907, 95% CI = 0.47–0.48). The mosquitoes collected belonged to the Anopheline, Culicine, and *Mansonia* genera. Regarding the mosquito genera, only Culicine mosquitoes were more abundant in the 2018 sampling year (6614/23,907, 95% CI = 0.27–0.28) compared to 2017 (5856/26,415, 95% CI = 0.22–0.23). Among the Anophelines were *An. gambiae* s.l. (93.2%), *An. pharoensis* (4.6%), *An. funestus* (2.1%), and *An. rufipes* (0.1%) (Table 1).

**Table 1:** Abundance and spatiotemporal distribution of mosquitoe genera and *Anopheles gambiae* sibling species

Mosquitoes	2017									
	Dry					Wet				
	Anyakpor	Dodowa	Dwease	Kpalsogu	Pagaza	Anyakpor	Dodowa	Dwease	Kpalsogu	Pagaza
Anopheline	1118	419	474	2,016	3	880	4858	1845	4203	2336
Culicine	1592	543	7	554	8	2534	458	10	66	84
Mansonia	22	20	4	35	0	15	19	139	1966	156
Total	2732	982	485	2605	11	3429	5335	1994	6235	2576
Anopheline species										
*An. gambiae*	1088	419	473	1911	3	759	4844	1844	3616	2288
*An. funestus*	0	0	0	0	0	0	1	0	44	35
*An. pharoensis*	29	0	1	104	0	120	12	1	537	11
*An. rufipes*	1	1	0	1	0	1	1	0	6	2
Total	1118	420	474	2016	3	880	4858	1845	4203	2336

Mosquitoes	2018									
	Dry					Wet				
	Anyakpor	Dodowa	Dwease	Kpalsogu	Pagaza	Anyakpor	Dodowa	Dwease	Kpalsogu	Pagaza
Anopheline	2013	402	848	389	183	1625	2054	442	1984	5241
Culicine	3960	327	8	86	6	1778	1778	14	69	59
Mansonia	0	25	20	611	3	37	39	147	979	251
Total	5973	754	876	1086	192	3440	3871	603	3032	5551
Anopheline species										
*An. gambiae*	1994	402	848	285	183	1253	2046	384	1737	4678
*An. funestus*	0	0	0	1	0	0	0	58	14	537
*An. pharoensis*	17	0	0	78	0	371	7	0	232	25
*An. rufipes*	2	0	0	25	0	1	1	0	1	1
Total	2013	402	848	389	183	1625	2054	442	1984	5241

Throughout the combined period of the study, abundance of *An. gambiae* s.l. varied significantly amongst the study sites (χ^2^ = 213.404; *df* = 4; *P* = 0.0001). The mean abundance of *An. gambiae* s.l. was highest in Pagaza (5.82, 95% CI = 5.43–6.24) and Dwease had the least (2.77, 95% CI = 2.65–2.90) (Additional file 1: Table S1). Overall, *An. gambiae* s.l. were more abundant in the rainy season (75.5%) than in the dry season (24.5%) during both sampling years (Z = − 36.037; *P* < 0.0001) (Additional file 1: Table S1). However, in Anyakpor and Dwease, more *An. gambiae* s.l. were collected in the dry season. Compared to the dry season, the mean abundance of *An. gambiae* s.l. was four-fold higher during the rainy season (B = 4.15, 95% CI = 3.916–4.381, *P* = 0.0001). Relatively fewer *An. gambiae* s.l. mosquitoes were collected in 2018 compared to the 2017 sampling year (B = − 0.871, 95% CI =  − 1.103 to − 0.0639, *P* = 0.0001) (Additional file 1: Table S3).

During both sampling years, almost all *An. funestus* were collected in the rainy season (B = 0.117, 95% CI = 0.15–0.20, *P* < 0.001) compared to the dry season (Additional file 1: Table S4). Unlike *An. gambiae* s.l., more *An*. *funestus* were collected during the 2018 sampling period (B = 0.136, 95% CI = 0.11–0.16, *P* < 0.001) than in the 2017 sampling period (Additional file 1: Table S4).

The most predominant species sampled were *An. pharoensis* [2017 n = (815/908); 2018 n = (730/1,371)], followed by *An. funestus* [2017 n = (80/908), 2018 n = (610/1371)], and then *An. rufipes* [2017 n = (13/908), 2018 n = (31/1371)]. More other Anopheline species were collected during the rainy season [2017 (*An*. *pharoensis* = 681/908, *An*. *funestus* = 80/908; *An*. *rufipes* = 10/908); 2018 (*An. pharoensis* = 635/1371, *An. funestus* = 609/1371, *An. rufipes* = 4/1371] compared to the dry season [2017 (*An*. *pharoensis* = 134/908; *An*. *funestus* = 0/908; *An*. *rufipes* = 3/908); 2018 (*An*. *pharoensis* = 95/1371, *An*. *funestus* = 1/1371, *An*. *rufipes* = 27/1371)].

### Indoor and outdoor distribution of vectors

Overall, more *An. gambiae* s.l. were collected indoors (51.1%; 15,866/31,055) than outdoors (48.9%; 15,189/31,055) (*Z* = 2.410; *P* = 0.0160) (Table 2, Additional file 1: Table S1). Contrarily, a total of 59.3% (409/690) *An. funestus* mosquitoes were collected outdoors while 40.7% (281/690) were collected indoors. There was a non-significant decrease in outdoor biting in *An. gambiae* s.l. (B = − 0.175, 95% CI = − 0.41 to 0.06 *P* = 0.140); however, the abundance of *An. funestus* was slightly increased outdoors (B = 0.033, 95% CI = 0.01–0.06, *P* = 0.005) compared to indoors (Additional file 1: Table S4).

**Table 2:** Biting location of *Anopheles gambiae* s.l. and *An*. *funestus*

Biting location	2017									
	Dry					Wet				
	Anyakpor	Dodowa	Dwease	Kpalsogu	Pagaza	Anyakpor	Dodowa	Dwease	Kpalsogu	Pagaza
*An. gambiae* s.l.										
Indoor	603	168	239	961	2	406	1956	1006	1807	1163
Outdoor	485	251	234	950	1	353	2888	838	1809	1125
Total	1088	419	473	1911	3	759	4844	1844	3616	2288
*An. funestus*										
Indoor	0	0	0	0	0	0	1	0	19	20
Outdoor	0	0	0	0	0	0	0	0	25	15
Total	0	0	0	0	0	0	1	0	44	35

Biting location	2018									
	Dry					Wet				
	Anyakpor	Dodowa	Dwease	Kpalsogu	Pagaza	Anyakpor	Dodowa	Dwease	Kpalsogu	Pagaza
*An. gambiae* s.l.										
Indoor	1212	213	419	155	108	720	1145	204	901	2478
Outdoor	782	189	429	130	75	533	901	180	836	2200
Total	1994	402	848	285	183	1253	2046	384	1737	4678
*An. funestus*										
Indoor	0	0	0	0	0	0	0	27	8	206
Outdoor	0	0	0	1	0	0	0	31	6	331
Total	0	0	0	1	0	0	0	58	14	537

**Fig. 2:**
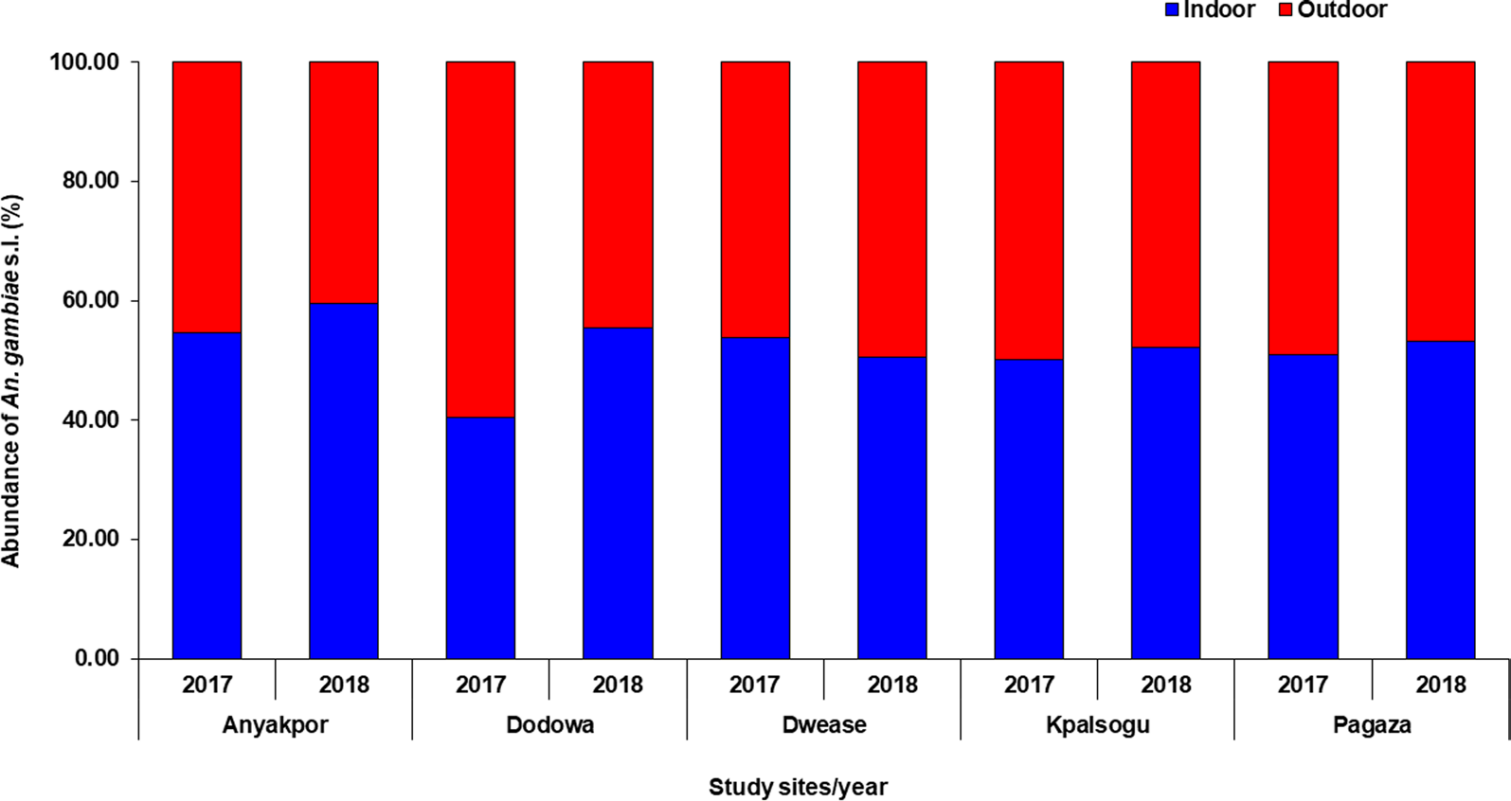
Biting behaviour of *Anopheles* *gambiae* s.l.

In 2017, 48.2% (8,311/17,245; 95% CI = 0.47–0.49) *An. gambiae* s.l. were collected indoors, and 54.7% (7555/13,810, 95% CI = 0.54–0.56) in 2018 as shown in Fig. 2, at all the study sites. The abundance of indoor-biting *An. gambiae* s.l. increased in the 2018 sampling period except in Dwease in the forest area where the abundance of indoor *An. gambiae* s.l. reduced from 53.7% (1245/2317, 95% CI = 0.52–0.56) in 2017 to 50.6% (623/1232, 95% CI = 0.48–0.53) 2018. During the 2017 sampling period, more *An. gambiae* s.l. were collected indoors in all sites, except in Dodowa where more were collected outdoors (59.6%; 3139/5263, 95% CI = 0.58–0.61) than indoors (40.4%; 2124/5263, 95% CI = 0.39–0.42) (Table 2, Fig. 2). During the 2017 sampling period, an equal number of *An. funestus* were collected both indoors (5%, 40/80) and outdoors (5%, 40/80) collections. However, in the 2018 sampling period, more *An. funestus* were collected outdoors (60.5%, 369/610) than indoors (39.5%, 241/610). Moreover, more *An. funestus* were collected (57.1%, 20/35) in indoor collection in Pagaza during the 2017 sampling period but during the 2018 sampling period more were collected outdoors (61.6%, 331/537). However, in Kpalsogu 43.2% (19/44) of *An. funestus* were collected during the 2017 sampling year and 53.3% (8/15) during the 2018 sampling year (Table 2).

### Species discrimination in the *An. gambiae* complex

**Fig. 3:**
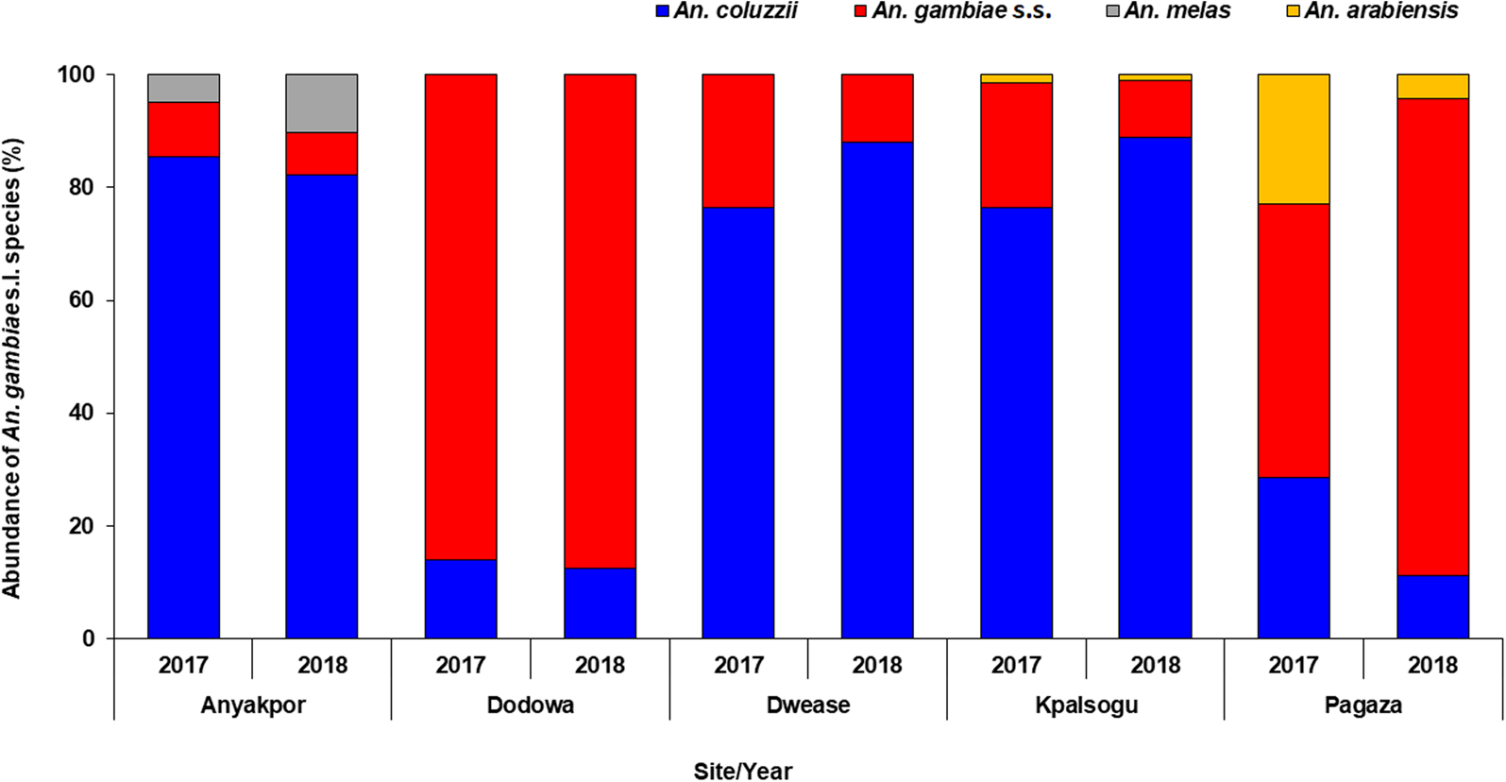
Distribution of *Anopheles* species according to study site

A subsample of 1670 *An. gambiae* s.l. from all the study sites was randomly selected and used to discriminate the sibling species: *An. coluzzii* 55.9% (935/1670), *An. gambiae* s.s. 39.5% (659/1670), *An. arabiensis* 2.3% (39/1670), and *An. melas* 2.2% (37/1670). Overall, more *An*. *coluzzii* were collected in all the ecological zones, except in the Sahel savannah zone, where the species were dominated by the *An*. *gambiae* s.s. [Sahel (*An*. *gambiae* s.s. = 323/1670; *An*. *coluzzii* = 298/1670; *An Arabiensis* = 39/1670; *An*. *melas* = 0/1670); coastal (*An*. *gambiae* s.s. = 288/1670; *An*. *coluzzii* = 432/1670; *An Arabiensis* = 0/1670; *An*. *melas* = 37/1670); forest (*An*. *gambiae* s.s. = 48/1670; *An*. *coluzzii* = 205/1670; *An. Arabiensis* = 0/1670; *An*. *melas* = 0/1670)] (Table 3, Fig. 3).

**Table 3:** Species discrimination of *Anopheles gambiae* s.l. per site

*An. gambiae* species	2017									
	Anyakpor		Dodowa		Dwease		Kpalsogu		Pagaza	
	Indoor	Outdoor	Indoor	Outdoor	Indoor	Outdoor	Indoor	Outdoor	Indoor	Outdoor
*An. arabiensis*	0	0	0	0	0	0	3	0	0	24
*An. coluzzii*	103	73	11	10	63	54	87	72	9	21
*An. gambiae* s.s.	5	15	63	65	11	25	17	29	41	10
*An. melas*	2	8	0	0	0	0	0	0	0	0
Total	110	96	74	75	74	79	107	101	50	55

*An. gambiae* species	2018									
	Anyakpor		Dodowa		Dwease		Kpalsogu		Pagaza	
	Indoor	Outdoor	Indoor	Outdoor	Indoor	Outdoor	Indoor	Outdoor	Indoor	Outdoor
*An. arabiensis*	0	0	0	0	0	0	1	0	9	2
*An. coluzzii*	137	81	11	6	44	44	47	33	20	9
*An. gambiae* s.s.	13	7	64	56	6	6	5	4	108	109
*An. melas*	10	17	0	0	0	0	0	0	0	0
Total	160	105	75	62	50	50	53	37	137	120

**Fig. 4:**
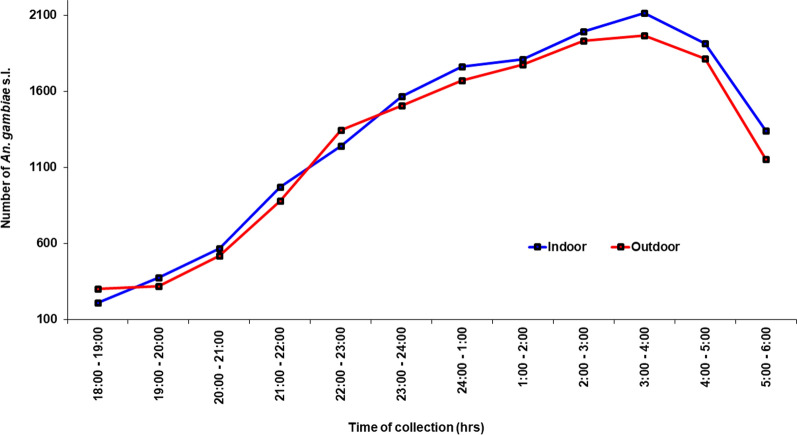
Biting times of *Anopheles* *gambiae* s.l.

The composition and distribution of these species differed significantly by study sites (*χ*^2^ = 967.48, df = 12, *P* < 0.001) and year (*χ*^2^ = 256.67, df = 3, *P* < 0.001). *Anopheles coluzzii* was the most abundant in Anyakpor (83.7%, 394/471), Dwease (81.0%, 205/253), and Kpalsogu (80.2%, 239/298) while *An. gambiae* s.s. was the most abundant in Dodowa (86.7%, 248/286) and Pagaza (74.0%, 268/362) respectively. All the *An. melas* collected during the study were only from the coastal savannah site of Anyakpor (7.9%, 37/471). All *Anopheles arabiensis* were from Sahel savannah sites of Kpalsogu (1.3%, 4/298) and Pagaza (9.7%, 35/362) (Table 3, Fig. 4).

### Biting times of *An. gambiae* s.l. and *An. funestus* in the study sites

*Anopheles gambiae* s.l. were found to bite the most during the late evening (LE) (66.6%; 20,685/31,055), followed by the early morning (EM) (20.1%; 6228/31,055), and less during the early evening (EE) (13.3%; 4142/31,055). This was the same pattern for both indoor [LE: (66.1%, 10,490/15,866); EM: (20.5%, 3255/15,866); EE: (11.4%, 2121/18,566)] and outdoor biting [LE: (67.1%, 10,195/15,189); EM: (19.6%, 2973/15,189), EE: (14.0%, 2021/15189)] (Fig. 4).

**Fig. 5:**
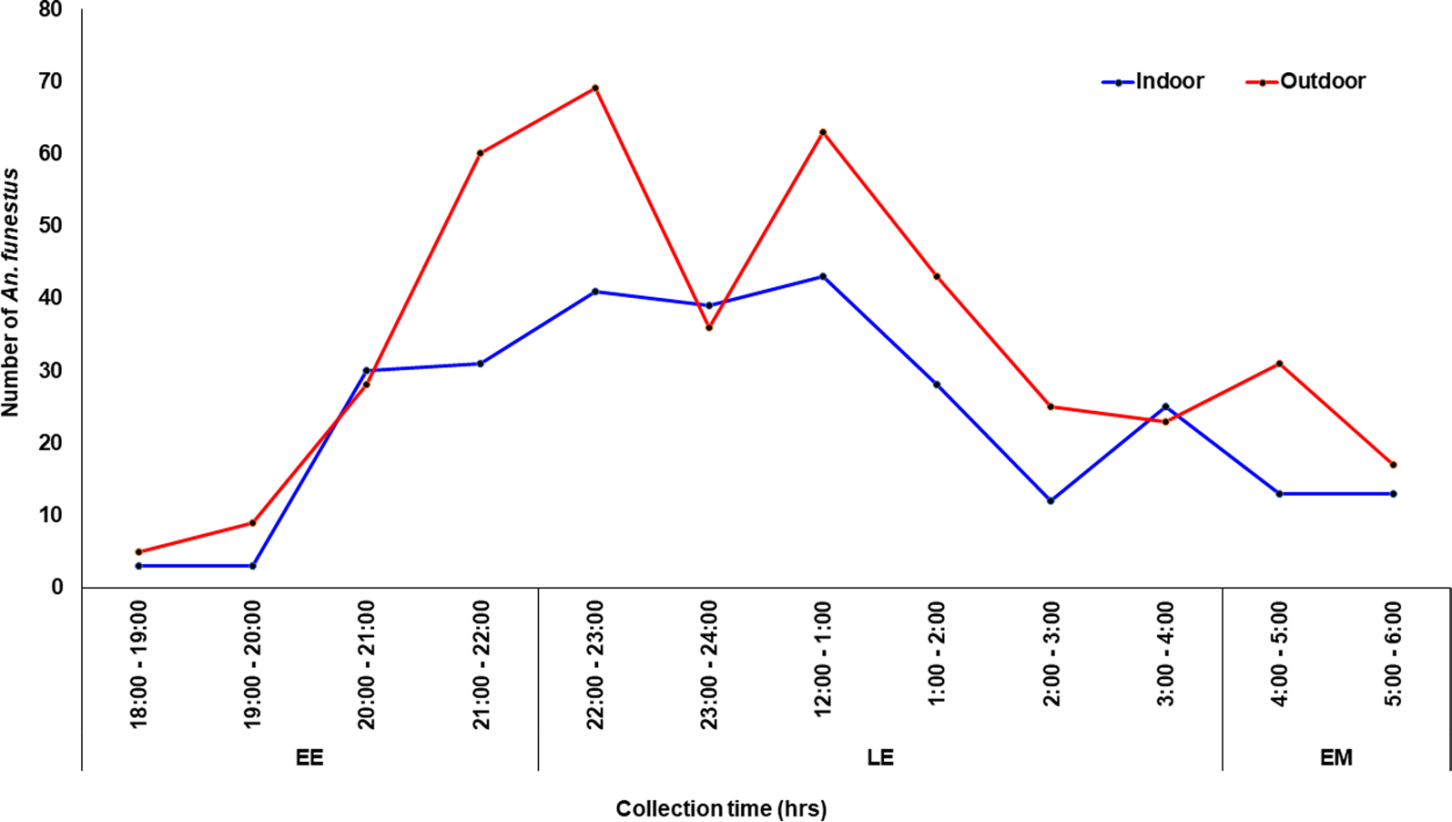
Biting times of *Anopheles* *funestus*

*Anopheles funestus* were found to bite mostly during the late evening (64.78%; 447/690) followed by the early evening (24.5%, 169/690) and early morning (10.7%; 74/690). This observed biting behaviour was similar for both indoor- [LE: (66.9%, 188/281); EE: (23.8%, 67/281); EM: (9.3%, 26/281)] and outdoor-biting *An*. *funestus* mosquitoes [LE: (63.3%, 259/409); EE: (24.9%, 102/409); EM: (11.7%, 48/409)] (Fig. 5). Compared to early evening biting activity, *An. gambiae* s.l. preferred to bite more in the late evenings (B = 3.723, 95% CI = 3.46–3.98, *P* = 0.000) and the early mornings (B = 3.209, 95% CI = 2.86–3.56, *P* = 0.000) (Additional file 1: Table S3). However, *An. funestus* biting activity increased significantly only in the late evenings compared to the early evenings (B = 0.050, 95% CI = 0.02–0.08, *P* < 0.0001) (Additional file 1: Table S4).

Regarding the species of *An. gambiae* s.l., *An. coluzzii* and *An. melas* had a different biting pattern from *An. gambiae* s.s. and *An. arabiensis*. *Anopheles coluzzii* preferred late evening feeding (48.0%, 449/935) followed by early morning (26.5%, 248/935) and early evening (25.5%, 238/935) feeding. *Anopheles melas* on the other hand preferred to bite in the early evening (43.2%, 16/37) followed by the late evening (40.5%, 15/37) and early morning (16.2%, 6/37). *Anopheles gambiae* s.s. preferred to bite in the late evening (54.5%, 359/659) followed by the early evening (25.0%, 165/659) and the early morning (20.5%, 135/659). *Anopheles arabiensis* preferred late evening (41.0%, 16/36) biting followed by early evening (33.3%, 13/36) and early morning (25.6%, 10/36) biting.

### Insecticide resistance genotypes in *An. gambiae *s.l.

*Anopheles gambiae* s.l. samples were genotyped for the presence of Vgsc-1014S and 1014F mutations as well as the G119S mutation. The Vgsc-1014S mutation was not detected in any mosquito for this study; however, the frequency of the Vgsc-1014F mutation was slightly varied in indoor-biting mosquitoes (54.9%) compared with those biting outdoors (45.1%). Overall, Vgsc-1014S mutation frequency in *An. melas* was 87.1%, whereas that of *An*. *arabiensis* was 50% (Table 4).

**Table 4:** Frequency distribution of *kdr L1014F* and *Ace-1 G119S* mutation

	N	*Kdr* L1014F	n	F (*kdr*)	N	*Ace* 1 G119S	n	F (*Ace-1*)
Indoor	873	RR	328	0.6	870	RR	178	0.5
		RS	338			RS	579	
		SS	207			SS	113	
Outdoor	752	RR	266	0.6	777	RR	120	0.5
		RS	302			RS	512	
		SS	184			SS	145	
Species								
*Anopheles arabiensis*	36	RR	13	0.5	39	RR	2	0.4
		RS	10			RS	30	
		SS	13			SS	7	
*An. coluzzii*	908	RR	300	0.5	924	RR	187	0.5
		RS	363			RS	591	
		SS	245			SS	146	
*An. gambiae* s.s.	646	RR	255	0.6	648	RR	96	0.5
		RS	258			RS	451	
		SS	133			SS	101	
*An. melas*	35	RR	26	0.9	36	RR	13	0.6
		RS	9			RS	19	
		SS	0			SS	4	

F(kdr) = 2RR + RS/2n Ahadji-Dabla 2019. F: allelic frequency, N = number of samples tested, *n* = total number of samples positive for a specific genotype

Similarly, the frequency of the G119S mutation in *An. gambiae* s.l. varied in indoor host-seeking mosquitoes (52.9%) compared with outdoor-biting mosquitoes (47.1%). Resistance mutation genotypes in the other *Anopheles* mosquitoes were *An*. *melas* 0.63 (30.1%) and *An. arabiensis* 0.44 (21.0%) (Table 4).

### Sporozoite infection rates in the sampled vectors

A total of 12,860 *An. gambiae* s.l. were pooled in groups of 20 into 643 pools and tested for *Plasmodium falciparum* circumsporozoite (CSP). Overall, 44 pools were positive for *P. falciparum* CSP—Anyakpor (*n* = 8), Dodowa (*n* = 7), Dwease (*n* = 10), Kpalsogu (*n* = 8), and Pagaza (*n* = 11)—giving an overall sporozoite rate of 0.1% (Table 5).

**Table 5:** Sporozoite rates in *Anopheles gambiae* s.l.

Community	Number per pools	Total pools	Positive pools	Dry season	Rainy season	Indoor	Outdoor	Sporozoite rate (SR)	Dry season SR	Rainy season SR	Indoor SR	Outdoor SR
Anyakpor	20	164	8	4	4	7	1	0.2	0.1	0.1	0.2	0.0
Dodowa	20	105	7	5	2	3	4	0.3	0.2	0.1	0.1	0.2
Dwease	20	103	10	5	5	6	4	0.5	0.2	0.2	0.3	0.2
Pagaza	20	131	11	4	7	6	5	0.4	0.2	0.3	0.2	0.2
Kpalsogu	20	140	8	4	4	3	5	0.3	0.1	0.1	0.1	0.2
		643	44									

^*^Sporozoite rate (SR) = [(no of positive pools)/(no of pools × maximal pool size)] × 100 Wei-Dong Gu 199

Regarding the individual study sites, sporozoite rate varied in indoor-collected mosquitoes compared to those collected outdoors except in Dodowa [(indoor (0.1%); outdoor (0.2%)] and Kpalsogu [(indoor (0.1%); outdoor (0.18%)] where outdoor-biting *An. gambiae* s.l. had a higher sporozoite rate.

Furthermore, all the study sites had similar sporozoite rates in both seasons as represented in Table 5 except in Dodowa where the sporozoite rate varied in the dry season (0.2%) compared to the rainy season (0.1%) and in Pagaza [rainy season (0.3%); dry season (0.2%)].

## Discussion

Evidence has shown that successful malaria elimination strategies require vector control intervention that target the changing vector behaviour [39]. It is, therefore, essential to monitor the changing vector behaviour and ecology in the era of increasing malaria intervention to reduce the high disease burden. This study investigated the biting behaviour, resistant gene genotyping, and spatiotemporal dynamics of malaria vectors in Ghana.

Findings from this study indicated that many malaria vectors were sampled during the 2017 sampling year, with a decline in vectors in the subsequent year. This may be because of the effectiveness of the vector control tools deployed in those areas or probably a reduction in breeding sites. Moreover, most vectors were collected during the rainy season for both sampling years, likely due to the availability of breeding habitats that facilitate oviposition by gravid females. However, the presence of mosquitoes in high abundance during the dry season in Anyakpor and Kpalsogu was likely due to the irrigated farming, which supports the breeding of vectors during the dry season [5, 29]. During the rainy season, these low-lying areas get flooded, therefore disrupting malaria vector breeding; however, during the dry season, the irrigation areas provide breeding habitats for continuous vector breeding. This implies that malaria transmission may be occurring in these areas year round [40, 41].

Malaria vectors in Africa have been efficient in malaria transmission largely because of their anthropophilic and endophilic nature [6, 42]. Therefore, knowledge of the biting behaviour in disease vectors is important to understand the role of the vectors in disease transmission and hence the deployment of effective control tools. In this study, the abundance of *Anopheles* mosquitoes biting indoors was relatively similar to the outdoor biting. This behavioural trait by the vectors was consistent for both sampling years. Compared to a study done in Ghana by Tuno et al. [43] in which the abundances of outdoor *An*. *gambiae* were 15% and 23% during the dry and rainy season, respectively, this study has shown a drastic increase in the outdoor-biting activity in *An*. *gambiae* s.l. According to Sherrard-Smith et al. (2019), mathematical models suggest that even the smallest changes in outdoor host-seeking activity of malaria vectors can have a substantial public health impact.

Larger increases in outdoor biting behaviour lead to reduced effectiveness of LLINs [44]. The shift in biting behaviour may be due to selective pressure mounted by the use of LLINs and IRS. Because LLINs and IRS are indoor based, increase in outdoor-biting mosquitoes may indicate possible outdoor malaria transmission, showing the need for outdoor vector interventions [45, 46]. Historically, the large-scale use of LLINs and IRS as led to an increase in the abundance of outdoor-biting vectors [23]. The high densities of outdoor-biting *An*. *gambiae* contribute to the persistence of malaria in the Sahel savannah area despite LLIN and IRS interventions [23]. The presence of outdoor-feeding mosquitoes limits the effectiveness of these interventions [23, 47] and may be of major public health concern. It will be important for vector control strategies targeting both indoor and outdoor malaria vectors to be introduced in these areas.

Overall, *An*. *coluzzii* was the most abundant vector; however, *An*. *gambiae* s.s. was the predominant species present in the costal savannah and forest zone. This is indicative that the primary malaria vectors are well established across all ecological zones; hence, constant surveillance and strengthening of control strategies are essential. These findings corroborate those of Hinne et al. [29] whose study was done in similar study sites and reported high abundance of *An. coluzzii* compared to *An. gambiae* s.s. in these areas [29].

Findings from this study showed that both indoor and outdoor *An*. *gambiae* s.l. preferred to bite late in the night when people were asleep. Peak biting activity in the late night occurs because household members begin to rise as early as 03:00 h to begin morning chores including fetching water and firewood, feeding animals, cooking, bathing, and preparing for market days. Outdoor sleeping is also a major factor contributing to peak outdoor biting in the late evenings. People sleep outside during the early nighttime because of high temperatures in the rooms and wait till about 02:00 h [47] when their rooms are cool enough to sleep indoors. During the dry season in the Sahel savannah areas, some people spend the entire night sleeping outdoors because their rooms become extremely warm. Other outdoor activities such as funerals, church activities, and trading are reasons for people to stay outside in the late evening [47]. This finding corroborates a study in Uganda that reported that the peak biting time for *An*. *gambiae* s.l. was between 23:00 and 5:00 h [14]. The biting behavioural activity observed in *An*. *melas* and *An*. *arabiensis* in correlation to the vector densities observed for both indoor and outdoor settings could have major public health implications, because with the level of resistance and sporozoite rate observed in the malaria vectors, there could be possible transmission of malaria outdoors (residual malaria). Malaria vector feeding and resting behaviours are likely to change to maximize available feeding opportunities. *Anopheles melas* and *An*. *arabiensis* preferred to bite their host outdoors compared to indoors, whereas *An*. *coluzzii* and *An*. *gambiae* s.s. preferred indoor biting.

The frequency of *kdr* mutations was very high but similar in outdoor- and indoor-biting mosquitoes. This could be because of frequent exposure to sub-lethal doses of insecticides for public health use, i.e. IRS, aerosol sprays, and LLINs used in houses, use of pesticides in agriculture [48], other volatiles in outdoor settings, and other factors that may be associated with insecticide resistance. Furthermore, this finding may also imply that the mosquito population from the study sites can resist the presence of insecticides employed for vector control and may lead to increased human-vector contact and malaria transmission in the region despite the high LLIN coverage. The Vgsc-1014F mutation has been found to be strongly associated with pyrethroid resistance in West Africa; consequently, their presence in indoor-biting mosquitoes may be of particular concern [49]. The presence of Vgsc-1014F mutation in a mosquito population is a reliable marker of both high individual target-site resistance and pyrethroid-resistance prevalence [50, 51]. Studies have shown a relationship between the spread of Vgsc-1014F alleles with the use of LLINs [52, 53]. The lowest frequency of Vgsc-1014F was found in *An. arabiensis*, a gene that confers target site resistance, which could be explained by the biting behaviour of this mosquito species. *Anopheles arabiensis* prefers to bite and rest outdoors, and this could have limited their exposure to the insecticides used in vector control.

The results from the study showed that the frequency of G119S from the mosquito population was higher in the indoor than outdoor mosquito population from all the study sites. The highest frequency of G119S was observed in *An. melas* and the lowest in *An. arabiensis*. That high frequency of *Ace*-1 mutation observed in Anyarkpor may be due to the frequent use of pesticides in agricultural activities in the area and exposure to malaria vectors since most of these pesticides contain the same active ingredients as insecticides used for public health control of malaria vectors [54–56]. This may imply that vector control management tools may fail in such areas and requires careful monitoring. A similar observation was made in southern Ghana by Essandoh et al. [25], who reported that high prevalence of resistance in malaria vectors was consistent with agriculture-driven selection.

Sporozoite rates determined during the study were relatively similar for both sampling seasons (dry and rainy) whereas relatively similar for indoor and outdoor sampling. This finding suggests that malaria transmission did not change between the seasons. However, sporozoite rate was not determined according to species and year of sampling from the various study sites. This was because blood-fed mosquito samples were pooled according to study sites for the determination of sporozoite rate, and this was a limitation to our study. Higher sporozoite rates were recorded in indoor mosquitoes compared to outdoor-biting mosquitoes from all the study sites except in Dodowa and Kpalsogu where the sporozoite rates were higher in mosquitoes collected outdoors than those from indoors. This observation was suggestive of outdoor malaria transmission (residual malaria), and it is important for vector control tools to be implemented to target outdoor-biting mosquitoes as well. The infection rates found in the indoor-biting mosquitoes could suggest ongoing malaria transmission regardless of vector control tools employed in the study sites.

## Conclusions

This study revealed that *An. gambiae* s.l. were more abundant indoors across all ecological zones of Ghana. Furthermore, the abundance of *Anopheles* mosquitoes and frequency of *kdr* mutations were similar in both indoor- and outdoor-biting mosquitoes. However, the frequency of G119S from the mosquito population was higher in the indoor than outdoor mosquito population from all the study sites. Higher sporozoite rates were recorded in outdoor mosquitoes in Dodowa and Kpalsogu. There is thus an urgent need for a supplementary malaria control intervention to control outdoor-resting and -biting mosquitoes as the current tools only target indoor-resting and -biting mosquitoes. Continued surveillance of vector behaviours is recommended to help in the control of malaria.

### Supplementary Information

**Additional file 1.Table S1.** Univariate analysis of sampling parameters on An. gambiae s.l. abundance.Univariate analysis of sampling parameters on *An. funestus* abundance**Table S2.** Univariate analysis of sampling parameters on *An. funestus* abundance. **Table S3.** Generalized linear mixed model of the effect of sampling parameters on *An. gambiae* s.l. abundance. **Table S3.** Generalized linear mixed model of the effect of sampling parameters on *An. funestus* abundance.

## Data Availability

All datasets generated and/or analysed during this study are included in the manuscript.

## Abbreviations

WHO: World Health Organisation
LLINs: Long lasting insecticide-treated nets
IRS: Indoor residual sprays
PMI: President Malaria Initiative
Ace-1: Acetylcholinesterase 1
kdr: Knockdown resistance
HLC: Human landing catch
EE: Early evening
LE: Late evening
EM: Early morning
PCR: Polymerase chain reaction
DNA: Deoxyribonucleic acid
PCR-RFLP: Polymerase chain reaction-restriction fragment length polymorphism
Vgsc: Voltage-gated sodium channel
SNP: Single nucleotide polymorphism
G: Glycine
L: Leucine
F: Phenylalanine
S: Serine
RR: Homozygote resistant
RS: Heterozygote resistant
SS: Homozygote susceptible
N: Number of samples tested
n: Total number of positive samples
NMIMR: Noguchi Memorial Institute for Medical Research
CSP: Circumsporozoite protein
