# Supplementary material for: Biting behaviour, spatio-temporal dynamics, and the insecticide resistance status of malaria vectors in different ecological zones in Ghana
Source: Parasit Vectors. 2024 Jan 9;17:16. doi: 10.1186/s13071-023-06065-9 (PMC10775458; doi:10.1186/s13071-023-06065-9)
Supplement: Supplementary file 1 — Additional file 1.Table S1. Univariate analysis of sampling parameters on An. gambiae s.l. abundance.Univariate analysis of sampling parameters on An. funestus abundanceTable S2. Univariate analysis of sampling parameters on An. funestus abundance. Table S3. Generalized linear mixed model of the effect of sampling parameters on An. gambiae s.l. abundance. Table S3. Generalized linear mixed model of the effect of sampling parameters on An. funestus abundance. [file 13071_2023_6065_MOESM1_ESM.docx]

**Supplementary Tables**

| **Variable** | **Level** | **Mean *An. gambiae* abundance** | **CI** | **Statistics** | **Sig** |
| --- | --- | --- | --- | --- | --- |
| Season | Dry | 2.64 | 2.54 – 2.74 | Z = -36.037 *p* = 0.0000 | *** |
|  | Rainy | 5.13 | 4.97 – 5.31 |  |  |
| Biting behaviour | Indoor | 4.14 | 3.99 – 4.29 | Z = 2.14  *p* = 0.016 | ** |
|  | Outdoor | 3.80 | 3.66 – 3.95 |  |  |
| Site | Anyakpor | 3.30 | 3.12 – 3.48 | χ^2^ = 213.404  df = 4  *p* = 0.0001 | *** |
|  | Dodowa | 4.43 | 4.14 – 4.73 |  |  |
|  | Dwease | 2.77 | 2.65 – 2.90 |  |  |
|  | Kpalsogu | 4.42 | 4.19 – 4.65 |  |  |
|  | Pagaza | 5.82 | 5.43 – 6.24 |  |  |

**S1a: Univariate analysis of sampling parameters on *An. gambiae* s.l. abundance**

**** p<0.01, ** p<0.05, * p<0.1*

**S1b**

| **Site** | **Biting behaviour** | **Mean *An. gambiae* abundance** | **CI** | **Statistics** | **Sig** |
| --- | --- | --- | --- | --- | --- |
| Anyakpor | Indoor | 3.75 | 3.47 – 4.06 | Z = 3.402  *p* = 0.0007 | *** |
|  | Outdoor | 2.88 | 2.67 – 3.11 |  |  |
| Dodowa | Indoor | 4.33 | 3.96 – 4.74 | Z = -0.357  *p* = 0.7212 |  |
|  | Outdoor | 4.53 | 4.09 – 5.00 |  |  |
| Dwease | Indoor | 2.94 | 2.76 – 3.13 | Z = 1.332  *p* = 0.7828 |  |
|  | Outdoor | 2.61 | 2.44 – 2.78 |  |  |
| Kpalsogu | Indoor | 4.49 | 4.17 – 4.83 | Z = 0.554  *p* = 0.5798 |  |
|  | Outdoor | 4.34 | 4.02 – 4.68 |  |  |
| Pagaza | Indoor | 5.96 | 5.40 – 6.58 | *p* = 0.4359  Z = 0.779 |  |
|  | Outdoor | 5.68 | 5.15 – 6.26 |  |  |

*** p<0.01, ** p<0.05, * p<0.1

**S2a: Univariate analysis of sampling parameters on *An. funestus* abundance**

| **Variable** | **Level** | **Mean *An. funestus* abundance** | **CI** | **Statistics** | **Sig** |
| --- | --- | --- | --- | --- | --- |
| Season | Dry | 1 |  | Z = -18.797  *p* = 0.0000 | *** |
|  | Rainy | 1.65 | 1.54 – 1.76 |  |  |
| Biting behaviour | Indoor | 1.56 | 1.42 – 1.71 | Z = -1.928  *p* = 0.0538 | * |
|  | Outdoor | 1.72 | 1.56 – 1.90 |  |  |
| Site | Anyakpor | 0 |  | χ^2^ = 619.971  df = 4  *p* = 0.0001 | *** |
|  | Dodowa | 1 |  |  |  |
|  | Dwease | 1.24 | 1.11 – 1.40 |  |  |
|  | Kpalsogu | 1.08 | 1.02 – 1.15 |  |  |
|  | Pagaza | 1.91 | 1.75 – 2.01 |  |  |

**S2b**

| **Site** | **Biting behaviour** | **Mean *An. funestus* abundance** | **CI** | **Statistics** | **Sig** |
| --- | --- | --- | --- | --- | --- |
| Anyakpor | Indoor | 0 | . |  |  |
|  | Outdoor | 0 | . |  |  |
| Dodowa | Indoor | . | . |  |  |
|  | Outdoor | . | . |  |  |
| Dwease | Indoor | 1.13 | 1.00 – 1.27 | Z = 1.332  *p* = 0.7828 |  |
|  | Outdoor | 1.40 | 1.13 – 1.72 |  |  |
| Kpalsogu | Indoor | 1.03 | 0.97 – 1.08 | Z = -0.158  *p* = 0.8741 |  |
|  | Outdoor | 1.14 | 1.02 – 1.27 |  |  |
| Pagaza | Indoor | 1.88 | 1.67 – 2.12 | Z = -2.603  *p* = 0.0092 | *** |
|  | Outdoor | 1.93 | 1.72 – 2.18 |  |  |

**S3: Generalized linear mixed model of the effect of sampling parameters on *An. gambiae* s.l. abundance**

| **Variable** | **Level** | **Coef. (B adj)** | **z** | **p>\|z\|** | **95%CI** | **Sig** |
| --- | --- | --- | --- | --- | --- | --- |
| Year | 2017 | 1 |  |  |  |  |
|  | 2018 | -0.871 | -7.35 | 0.000 | -1.10 – -0.634 | *** |
| Site | Anyakpor | 1 |  |  |  |  |
|  | Dodowa | 1.704 | 9.05 | 0.000 | 1.34 – 2.07 | *** |
|  | Dwease | -1.064 | -5.72 | 0.000 | -1.43 – -0.70 | *** |
|  | Kpalsogu | 1.599 | 8.49 | 0.000 | 1.23 – 1.97 | *** |
|  | Pagaza | 1.340 | 7.12 | 0.000 | 0.97 – 1.71 | *** |
| Season | Dry | 1 |  |  |  |  |
|  | Rainy | 4.149 | 35.00 | 0.000 | 3.95 – 4.38 | *** |
| Biting location | Indoor | 1 |  |  |  |  |
|  | Outdoor | -0.175 | -1.48 | 0.140 | -0.41 – 0.06 |  |
|  |  |  |  |  |  |  |
| Biting time | Early Evening | 1 |  |  |  |  |
|  | Late Evening | 3.723 | 28.14 | 0.000 | 3.46 – 3.98 | *** |
|  | Early Morning | 3.209 | 18.08 | 0.000 | 2.86 – 3.56 | *** |
|  |  |  |  |  |  |  |

**** p<0.01, ** p<0.05, * p<0.1*

**S4: Generalized linear mixed model of the effect of sampling parameters on *An. funestus* abundance**

| **Variable** | **Level** | **Coef. (B adj)** | **z** | **p>\|z\|** | **95%CI** | **Sig** |
| --- | --- | --- | --- | --- | --- | --- |
| Year | 2017 | 1 |  |  |  |  |
|  | 2018 | 0.136 | 11.68 | 0.000 | 0.11 – 0.16 | *** |
|  |  |  |  |  |  |  |
| Site | Anyakpor | 1 |  |  |  |  |
|  | Dodowa | 0.001 | 0.04 | 0.971 | -0.04 – 0.04 |  |
|  | Dwease | 0.043 | 2.35 | 0.019 | 0.01 – 0.08 | ** |
|  | Kpalsogu | 0.038 | 2.08 | 0.038 | 0.00 – 0.07 | ** |
|  | Pagaza | 0.372 | 20.15 | 0.000 | 0.34 – 0.41 | *** |
|  |  |  |  |  |  |  |
| Season | Dry | 1 |  |  |  |  |
|  | Rainy | 0.177 | 15.21 | 0.000 | 0.15 – 0.20 | *** |
|  |  |  |  |  |  |  |
| Biting location | Indoor | 1 |  |  |  |  |
|  | Outdoor | 0.033 | 2.83 | 0.005 | 0.01 – 0.06 | *** |
|  |  |  |  |  |  |  |
| Biting time | Early Evening | 1 |  |  |  |  |
|  | Late Evening | 0.050 | 3.84 | 0.000 | 0.02 – 0.08 | *** |
|  | Early Morning | -0.008 | -0.46 | 0.645 | -0.04 – 0.03 |  |
|  |  |  |  |  |  |  |

**** p<0.01, ** p<0.05, * p<0.1*
